# Vertical matrix perovskite X-ray detector for effective multi-energy discrimination

**DOI:** 10.1038/s41377-022-00791-y

**Published:** 2022-04-21

**Authors:** Jincong Pang, Shan Zhao, Xinyuan Du, Haodi Wu, Guangda Niu, Jiang Tang

**Affiliations:** 1 Wuhan National Laboratory for Optoelectronics and School of Optical and Electronic Information, Huazhong University of Science and Technology, 430074 Wuhan, China; 2 Optical Valley Laboratory, 430074 Wuhan, China

**Keywords:** X-rays, Imaging and sensing

## Abstract

Multi-energy X-ray detection is sought after for a wide range of applications including medical imaging, security checking and industrial flaw inspection. Perovskite X-ray detectors are superior in terms of high sensitivity and low detection limit, which lays a foundation for multi-energy discrimination. However, the extended capability of the perovskite detector for multi-energy X-ray detection is challenging and has never been reported. Herein we report the design of vertical matrix perovskite X-ray detectors for multi-energy detection, based on the attenuation behavior of X-ray within the detector and machine learning algorithm. This platform is independent of the complex X-ray source components that constrain the energy discrimination capability. We show that the incident X-ray spectra could be accurately reconstructed from the conversion matrix and measured photocurrent response. Moreover, the detector could produce a set of images containing the density-graded information under single exposure, and locate the concealed position for all low-, medium- and high-density substances. Our findings suggest a new generation of X-ray detectors with features of multi-energy discrimination, density differentiation, and contrast-enhanced imaging.

## Abstract (web summary)

The vertical matrix perovskite X-ray detectors for multi-energy detection is reported, providing a new generation of X-ray detectors with features of multi-energy discrimination, density differentiation and contrast-enhanced.

## Graphical abstract

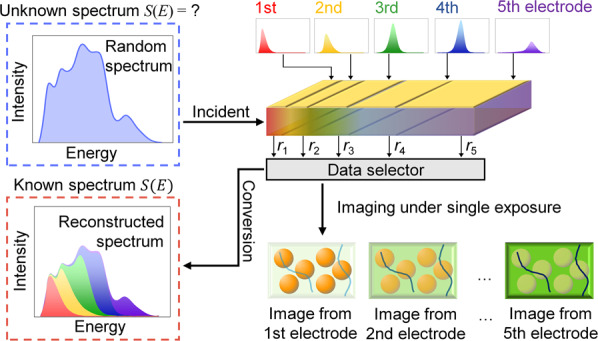

## Introduction

X-ray detection is widely used in medical imaging, security checking, radioactivity detection, industrial flaw inspection, and so on^1–5^. In this field, a long-term research direction is to develop dual or multi-energy X-ray detection for obtaining two or more sets of signals from high and low-energy X-ray^6–8^. This detection method is under intensive developments considering its great importance and superior difficulty. Such detectors are advantageous in distinguishing substances with different densities, enhancing imaging contrast between organic and inorganic items, as well as identifying softer matters (rubber, plastics and tissue). Furthermore, dual or multi-energy X-ray discrimination also enables the use of digital subtraction to extract images of different tissues at the same position, such as subtracting the bones to reveal the lungs on the chest^9–11^.

Currently, only dual-energy X-ray detection could be barely realized by altering the incident X-ray energy, *i.e*. exposing the object to X-ray sources twice with altered energy successively^7^. Under high-energy X-ray, the substances with high density, like metals and skeletons, could be detected, while under low-energy X-ray, the substances with both high density and low density (plastics and veins) could be imaged^12^. However, the major drawback is that the low-energy and high-energy images cannot perfectly overlap due to the temporal differences between the exposures, including cardiac and respiratory motion, as well as variation in contrast-agent concentration. Other drawbacks are that the radiation dose is inevitably increased during multiply X-ray exposures, and this method is hard to extend to multi-energy X-ray detection, due to the high requirement of rapid voltage switching of X-ray sources^11^.

To circumvent the above disadvantages, dual-layer detectors have been raised, where the top layer preferentially detects low-energy photons, and the bottom layer detects a filtered and therefore harder spectrum (high-energy photons)^11^. Nevertheless, only two layers still cannot achieve multi-energy X-ray detection, and the energy discrimination capability is rather low. We calculated the energy discrimination capability of the existing dual-layer detector and compared the performance with the quintuple-layer detector (see detailed discussion in Supporting Information, Tables S1 and S2). Additionally, dual-layer detectors always employ scintillators for indirect X-ray detection, which is limited in detection sensitivity and requires a high radiation dose for clear imaging^13^.

Recently lead halide perovskites are emerging as promising semiconductors for direct X-ray detection, with excellent properties of large X-ray stopping power, high sensitivity and low detection limit^14–16^. The X-ray detection sensitivity is on the order of 10^3^–10^5^ μC Gy_air_^−1^ cm^−2^ ^13,17^. The detection limit is as low as tens of nGy_air_ s^−1^, which is equivalent to hundreds of X-ray photons per second^18^. Such high sensitivity and low detection limit indicate that perovskite X-ray detectors could respond toward low photon fluence, and lay a foundation for multi-energy discrimination. By reducing the dose required for a single valid detected signal, perovskite detectors lessen the total dose required for multi-energy detection exponentially. However, as far as we are concerned, perovskite X-ray detectors with the capability of multi-energy X-ray discrimination have never been reported.

Herein we demonstrate, for the first time, the novel design of “vertical matrix” perovskite X-ray detectors for multi-energy detection. Different from the traditional plane detector, the term “vertical matrix” means that the detector array is aligned along the incident direction of X-ray rather than perpendicular to the incident direction. The principle is that X-ray photons with different energy have different attenuation depths within the vertical matrix, and low-energy photons deposit most of the energy in the shallow layers and high-energy photons in deeper layers. Thereby, the vertical matrix which consists of more-than-two-layers detector could restore the X-ray spectrum in detail. Experimentally, we indeed obtain the response conversion matrix of the vertical matrix detector with the aid of a machine learning algorithm. The conversion matrix is the key to the multi-energy discrimination and imaging process. For demonstration, we accurately derived the cut-off energy values for unknown X-ray sources. More importantly, the detector could produce a set of images containing the density-graded information under single exposure, and locate the concealed position for all low-, medium- and high-density substances. Taken together, our work theoretically and experimentally verifies that the novel design of vertical matrix perovskite detectors is promising for next-generation X-ray detection with features of multi-energy discrimination, density differentiation, and contrast-enhanced imaging.

## Results

Fundamentally, X-ray photons are attenuated in the detector following the exponential form, and the transmitted X-ray intensity (*T*) could be expressed as:

1 $$T = T_0{{{\mathrm{e}}}}^{ - \alpha (E)l}$$

where *T*_0_ is the incident X-ray intensity, *l* is the interaction depth, and *α* is the linear attenuation coefficient. Basically, *α*(*E*) is proportional to *Z*^4^*/E*^3^ when only considering the photoelectric effect, where *Z* is the average atomic number and *E* is the X-ray photon energy^19^. Thereby the low-energy photons within the wide X-ray spectrum exhibit high attenuation coefficient and deposit most of the energy in the shallow layer. In contrast, high-energy photons can penetrate deeper within the detector. Based on the distinguishing characteristics of low-energy and high-energy photons, the applicable energy range of the following analysis is not limited to the photoelectric effect, but also applies to Compton scattering, that is, from keV to MeV.

**Fig. 1:**
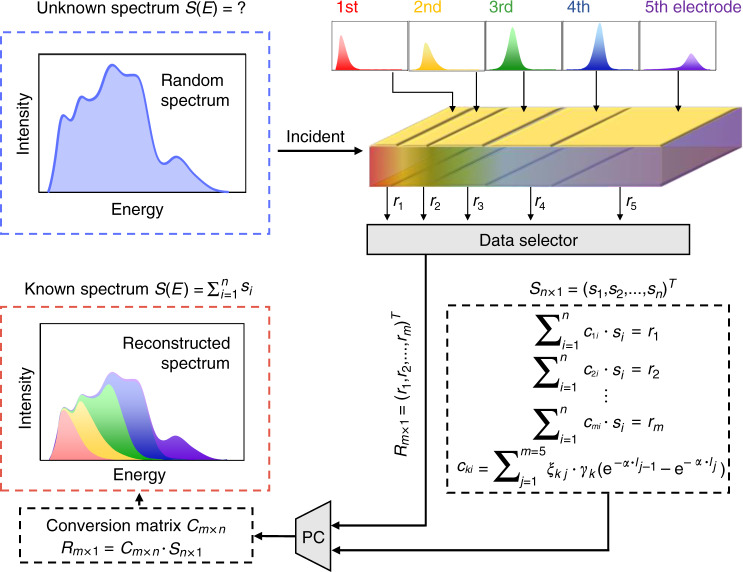
The schematic illustration of the working principle of multi-energy X-ray detection

Here we take advantage of this unique property of X-ray. Figure 1 shows the working principle. Along the incident X-ray path, we fabricate pairs of vertically-aligned electrodes on both sides of the crystal. As analyzed above, the response signals from the shallow layers are mainly from the low-energy X-ray photons, while those of the bottom layers are mainly from high-energy photons. For concise description, the layer sequence was labeled according to the electrode location from the left (the direction close to the X-ray source) to the right (the direction far away from the X-ray source), i.e. the uppermost or left layer is the 1st electrode and the nethermost or right layer is the *m*-th electrode for a total of *m* pairs of electrodes. One incident X-ray spectrum (*S*) can produce a series of response signals (*R*). The spectrum is divided into *n* segments as $S_{n \times 1} = (s_1,\;s_2{{{\mathrm{,}}}} \ldots, s_n)^T$ based on the energy. If *n* is approaching infinity, *s*_*i*_ is approximately monochromatic X-ray, $i \in \{ 1,2, \ldots ,n\}$. If *n* is a finite value, *s*_*i*_ represents a segmented energy range of X-ray. The response matrix (*R*_*m×*1_) could be expressed as $R_{m \times 1} = (r_1,\;r_2{{{\mathrm{,}}}} \ldots, r_m)^T$, where *r*_*k*_ is the response signal for the *k*-th pair of electrodes, $k \in \{ 1,2, \ldots ,m\}$. When ignoring the spacing between the neighboring electrodes, *r*_*k*_ could be described as follows:

2 $$r_k = \mathop {\sum}\limits_{i = 1}^n {\mathop {\sum}\limits_{j = 1}^m {\xi _{kj} \cdot \gamma _k \cdot \left[ {e^{ - \alpha (E_i)l_{j - 1}} - e^{ - \alpha (E_i)l_j}} \right] \cdot s_i} }$$

where *s*_*i*_ represents the intensity of X-ray photons with a given spectrum and *α*(*E*_*i*_) is the linear attenuation coefficient of them, *l*_*j*_ is the distance from the incident surface of the detector to the end of the *j*-th electrode, $j \in \{ 1,2,...,m\}$, *γ*_*k*_ describes the X-ray conversion efficiency, and *ξ*_*kj*_ is the charge collection efficiency within the width of the *j*-th electrode towards the *k*-th electrode. The coefficient, *ξ*_*kj*_ *γ*_*k*_, is decided by the bias voltage, carrier mobility-lifetime product and other factors of the detector. Thus, it is a fixed value for the certain detector under the given working state. It should be noted that for the charge collection efficiency term, *ξ*_*kj*_ is a δ function, if we ignore the charge lateral diffusion, dispersion of the photoelectron cloud, re-absorption of k-edge fluorescence and the curved distribution of electric field lines. The value of *ξ*_*kj*_ is 1 at *k* = *j* and 0 at *k* ≠ *j*. As discussed in Supporting Information, here we found that the crosstalk caused by interpixel charge diffusion, photoelectron cloud dispersion and re-absorption of k-edge fluorescence is negligible.

We sample one continuous X-ray spectrum (*S*) and make it discretized into *n* points. Then we get a spectral matrix containing spectral distribution information, as $S_{n \times 1} = (s_1,\;s_2{{{\mathrm{,}}}} \cdots, s_n)^T$. The relationship between spectral matrix *S*_*n*×1_ and detection response matrix *R*_*m*×1_ is as follows:

3 $$R_{m \times 1} = C_{m \times n} \cdot S_{n \times 1}$$

where *C*_*m*×*n*_ is the conversion matrix, $C_{m \times n} = (c_{ki})_{m \times n}$, and the element *c*_*ki*_ is written as follows, $k \in \{ 1,2,...,m\}$, $i \in \{ 1,2,...,n\}$:

4 $$c_{ki} = \mathop {\sum}\limits_{j = 1}^m {\xi _{kj} \cdot \gamma _k \cdot \left[ {e^{ - \alpha (E_i)l_{j - 1}} - e^{ - \alpha (E_i)l_j}} \right]}$$

Upon using the above detector for X-ray energy discrimination, the response signals are detected as $R_{m \times 1} = C_{m \times n} \cdot S_{n \times 1}$. Due to the theorem of Matrix Operations, that only a square matrix has an inverse matrix and the invertible matrix must be a square matrix, the conversion matrices *C*_*m*×*n*_ should be square matrices, i.e. *n* = *m*. In other words, the number of electrode pairs determines how many sampling points for the continuous X-ray spectrum. On this basis, we rewrite Eq. 3 as:

5 $$C_{m \times m}^{ - 1} \cdot R_{m \times 1} = S_{m \times 1}$$

where *C*_*m*×*m*_^−1^ is the inverse matrix of the conversion matrix of a certain vertical matrix detector. *C*_*m*×*m*_^−1^ is also unique for that detector. That means we can deduce the incident X-ray spectrum (*S*) in reverse by analyzing the response signals (*R*) of the detector.

Then we can use this detector for energy discrimination and multi-energy imaging applications. The key is to obtain the inverse matrix of the conversion matrix for the detector. Inspired by machine learning, we construct the data sets by using X-ray sources with known spectral shapes and recording the electrode response signals of the vertical matrix detector. The inverse conversion matrix is the model of the process, and it is important to make the errors as small as possible by optimizing the model. (Here, the model is a variable and non-fixed function, which refers to the result of the machine learning algorithm.) Firstly, we let the model learn the spectral matrix and response matrix of the Training Datasets, in order to find the relationship between their eigenvectors, without needing to know the values of every physical parameter. The maximum value of the percentage difference between the spectral sampling values and the model calculated values is used as the Loss Function (the judgment standard), and the gradient descent method is used as the optimization algorithm. Then, we verify the effectiveness of the model in the Validation Datasets, adjust hyper-parameters and get the optimized inverse conversion matrix.

We can change the driving voltages, the target anode and the attenuation sheets of the X-ray sources to vary Training Datasets and Validation Datasets. We demonstrate the concept by depositing five pairs of vertically-aligned electrodes onto one MAPbBr_3_ perovskite single crystal (PSC). When the incident spectrum is *S*_5×5_, the response signal can be obtained on each electrode, and the column vector *R*_5×5_ can be obtained after sampling. We can derive five segments of the incident X-ray spectrum according to the above analysis. The calculation matrix is as follows:

6 $$C_{5 \times 5}^{ - 1} \cdot R_{5 \times 5} = S_{5 \times 5}$$

where *R*_5×5_ = (*R*_1_, *R*_2_, *R*_3_, *R*_4_, *R*_5_) and *S*_5×5_ = (*S*_1_, *S*_2_, *S*_3_, *S*_4_, *S*_5_).

Here we chose MAPbBr_3_ PSC mainly due to its cuboid shape and convenient fabrication process. The surfaces of the crystals were carefully polished to achieve a parallel and smooth surface (Fig. S1). The X-ray attenuation curve of MAPbBr_3_ toward the X-ray energy range is shown in Fig. S2^20^. If the electrodes were fabricated with the equal thickness (or depth) and pitch size, there would be large fluctuations for the responses at electrodes with different numbers, since most of the energy would be deposited on the top layers of the detector (Fig. S3a). In order to avoid the large fluctuations and make small signals not to be submerged due to crosstalk, the electrode thickness was adjusted.

**Fig. 2:**
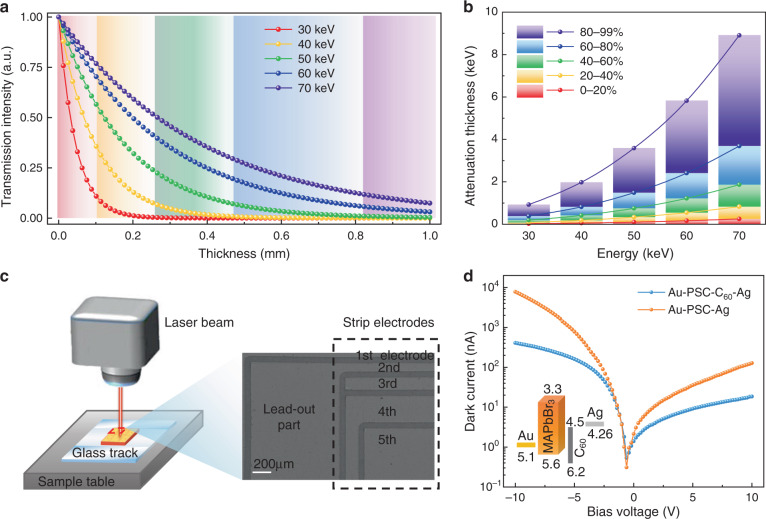
The design of the electrode structure for the vertical matrix detector. **a** The thickness-attenuation curve of MAPbBr_3_ under different X-ray energy. **b** The thickness required for every 20% X-ray intensity attenuation under different energy. **c** Schematic diagram of the strip electrodes etched by laser marking, and the SEM picture of them in the enlarged view. **d** *I*–*V* curves of Au–PSC–Ag device and Au–PSC–C_60_-Ag device in the darkness. The inset is the energy level diagram of Au–PSC–C_60_-Ag device

The electrode design of the vertical matrix detector involved in this article is developed for a specific energy range. We firstly supposed that the incident spectrum was a square spectrum from 30 to 70 keV, given that the driving voltage was at 70 kVp and the low-energy photons below 30 keV were blocked by the aluminum plate. This energy range is useful for applications like breast tomosynthesis and angiography. Each background color in Fig. 2a symbolizes the process of the intensity attenuation by 20%, until the intensity attenuates from 80% to 99% (purple background, without drawing the part whose thickness is >1 mm). The thickness required for the five-stage attenuation process by 20% of each energy intensity is shown in Fig. 2b. Here, the required attenuation thickness for the energy of 30, 40, 50, 60, and 70 keV is drawn as squares, and the calculated thickness values of other energy can be obtained in the entire spectral range. The lines in Fig. 2b connected by these series of values form the functions, *f*_*j*_(*E*), where *f*_*j*_ are the distances required for the five-stage attenuation of photons with certain energy, and *j* represents the five stages from 1st to 5th, which is equal to the electrode number as above. Then, the integral average values are calculated as:

7 $$Thickness_j = {\int}_{E_{min}}^{E_{max}} {\frac{{f_j(E) - f_{j - 1}(E)}}{{E_{max} - E_{min}}}dE}$$

where *E*_*min*_ is the minimum photon energy as 30 keV and *E*_*max*_ is the maximum photon energy as 70 keV. The results of each electrode thickness are shown in Table S3, representing that each electrode with such thickness will attenuate identical X-ray intensity under the square spectrum from 30 to 70 keV, i.e. each electrode will extract the similar amplitude of signals.

To fabricate the designed electrode patterns, we used a 1064 nm laser beam to etch the evaporated gold electrode patterns, as shown in Fig. 2c. A self-made glass parallel track was used to record the marking position, in order to ensure that the 1st electrode was close to the edge of the MAPbBr_3_ PSC and the patterns on both sides were aligned. The drawing of the laser beam processing is shown in Fig. S4a. The fabrication parameters have been slightly adjusted to avoid the destruction of perovskite by laser. Figure S4b shows the optical photo of the fabricated electrode pattern. In the enlarged view in Fig. 2c, the photo of scanning electron microscope is shown, among which we can clearly see the strip electrodes from top to bottom. The lead-out parts on the side were designed with a large square shape for simplifying the contact with probe systems. The size of laser processing traces is 60 μm, and this is the size between neighboring electrodes. The photos of energy dispersion spectrum in Fig. S4 prove that the gold electrode pattern has been successfully made and strip electrodes are isolated well from each other. More information about the fabricated electrode pattern can be found in Table S3. By the way, we used the fullerene layer to passivate the crystal surface and promote electron extraction^21^. The device has lower dark current and less ion migration compared with that without surface passivation in Fig. S5. The structure and energy band of the PSC device are shown in Fig. 2d, and the detector has a rectification ratio of 25. For X-ray detection, the device was driven in the positive bias for lower dark current.

**Fig. 3:**
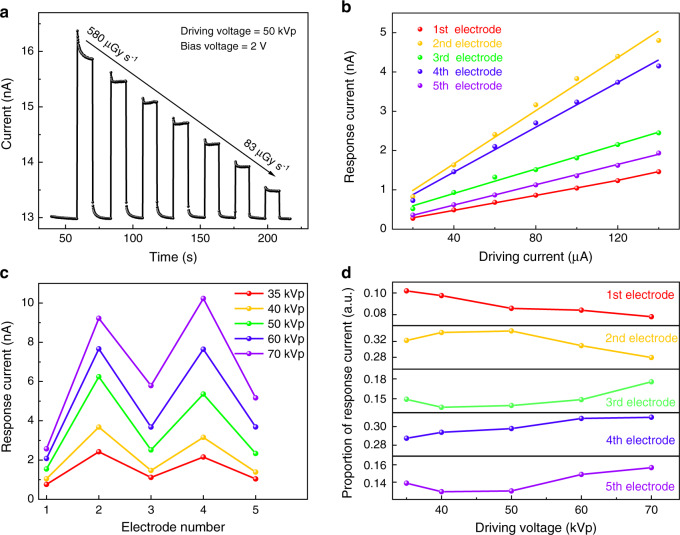
The X-ray response of the vertical matrix detector. **a** The current response curve of the 1st electrode toward different X-ray dose rates. **b** The linear response of the five pairs of electrodes. **c** The response current of the five pairs of electrodes under continuous multi-energy X-ray spectra with different driving voltages. **d** The proportion of the response current of each electrode under different driving voltages

Then we measured the performance of the MAPbBr_3_ PSC device according to the method shown in Fig. S6, in which the lead-out parts are covered by lead sheets to shield a part of the X-ray. Figure 3a shows the current response curve of the 1st electrode, which is exposed to the X-ray spectrum with the driving voltage of 50 kVp. The dark current is 13 nA with negligible baseline drift, and the response current is rapid and stable. The total dose rates are changed with the driving current in the range of 580 μGy_air_ s^−1^ to 83 μGy_air_ s^−1^ at equal intervals. Since in our design, the detector arrays are aligned along the incident direction of X-ray, and the conventional area sensitivity is not applicable to this kind of detector. Here we calculated the volume sensitivity of our detector. The volume sensitivity is 0.81 × 10^5^ μC Gy_air_^−1^ cm^−3^, which is close to previous works (2.76 × 10^5^ μC Gy_air_^−1^ cm^−3^ for BA_2_MA_2_Pb_3_I_10_, 0.25 × 10^5^ μC Gy_air_^−1^ cm^−3^ for MAPbI_3_)^22–24^. The detection limit is measured as 0.82 μGy_air_ s^−1^. Figure 3b shows that the response current of the five pairs of electrodes is proportional to the radiation dose rates for the same incident X-ray spectrum. The whole vertical matrix detector exhibits excellent linear response, of which the linear fitting coefficients *R*^2^ are 0.99911, 0.98981, 0.99295, 0.9925, and 0.99861 for the 1st to 5th electrode, respectively (Table S4). This good linear response to X-ray dose rate is extremely important for the multi-energy discrimination of unknown X-ray spectrum. We tested responses of the device under several different continuous multi-energy X-ray spectra via changing the driving voltage. The driving voltages were set at 35, 40, 50, 60, and 70 kVp, and the driving current of the X-ray tube was fixed at 100 μA. According to the principle of bremsstrahlung, the driving voltages affect the shapes of the spectra, especially the cut-off voltages^25^. The original response data can be found in Table S5. In Fig. 3c, it can be seen that the response current increases upon the driving voltages of the X-ray tube enhance. The five values of response current under each voltage fluctuate slightly, not exactly the same. It may be due to the slight difference between the actual electrode thickness and the designed thickness as Table S3. This makes X-ray photons deposited within the five segments slightly different. But the fluctuation is relatively small and does not affect the multi-energy X-ray detection too much. As we know, the signal crosstalk in the detector is an unavoidable problem and adjacent pixel signals will obscure the effective information of small-signal electrodes^26,27^. Only considering the carrier diffusion factor in crosstalk, the diffusion charge satisfies, *Q* = e*N*[1 − exp(−*V*Δ*y*^2^/4*V*_th_/*l*_total_^2^)], and the statistical mean square value of the carrier lateral diffusion distance is, Δ*y*^2^ = 2*l*_total_^2^(*V*_th_/*V*)^28^, where *V*_th_ = *k*_B_*T*/e is the thermal voltage, *V* is bias voltage, Δ*y* describes the location, *l*_total_ is the thickness of the detector as described above and *N* is the number of photogenerated carriers. The bigger Δ*y* and *N*, the greater the proportion of crosstalk of the signals from the reference electrode to surrounding electrodes. Therefore, the response signal of each electrode should be similar, instead of designing equal thickness electrodes as Fig. S3b, which avoids large crosstalk caused by the fluctuation and improves the X-ray energy discrimination capability. In Fig. 3d, we list the response signals of different electrodes towards different driving voltages of the X-ray tube. We define the proportion of one electrode to the whole spectrum via dividing the response signal of the given electrode by the summed signal of the five electrodes. The proportion of the response current is used to weigh how much energy is deposited within one electrode thickness. It should be noted that for the 1st and 2nd electrodes, the response proportion decreased as the driving voltage increased, which is due to that lower energy X-ray deposits its energy more in the shallow layers.

As mentioned above, the key is to calculate the inverse matrix of the conversion matrix for the detector. Here we use the data in Fig. 3c and get the response matrix of the MAPbBr_3_ detector as $R_{5 \times 5} = (R_{35},\;R_{40},\;R_{50},\;R_{60}{{{\mathrm{,}}}}\;R_{70})$, where

$$\begin{array}{l}R_{35} = (0.76,\;2.42,\;1.12,\;2.15,\;1.04)^T\\ R_{40} = (1.05,\;3.68,\;1.48,\;3.16,\;1.39)^T\\ R_{50} = (1.54,\;6.24,\;2.51,\;5.36,\;2.34)^T\\ R_{60} = (2.08,\;7.66,\;3.68,\;7.64,\;3.68)^T\\ R_{70} = (2.57,\;9.22,\;5.79,\;10.23,\;5.17)^T\end{array}$$

Each subscript of the element in matrix *R*_5×5_ represents the driving voltage of the corresponding X-ray energy spectrum. We used Monte Carlo simulation to obtain the theoretical energy spectra of the X-ray tube under given driving voltages, as shown in Fig. S7. More information about the modeling process can be found in Supporting Information. The elements in the spectral matrix *S*_5×5_ are discrete values of the incident spectra, which are sampled at 30, 40, 45 55, and 65 keV. The subscripts of those elements also represent the magnitude of the driving voltages, which are 35, 40, 50, 60, and 70 kVp. $S_{5 \times 5} = (S_{35},\;S_{40},\;S_{50},\;S_{60}{{{\mathrm{,}}}}\;S_{70}),$ is normalized as:

$$\begin{array}{l}S_{35} = (0.06,\;0.00,\;0.00,\;0.00,\;0.00)^T\\ S_{40} = (0.12,\;0.02,\;0.00,\;0.00,\;0.00)^T\\ S_{50} = (0.37,\;0.16,\;0.08,\;0.00,\;0.00)^T\\ S_{60} = (0.47,\;0.25,\;0.17,\;0.03,\;0.00)^T\\ S_{70} = (1.00,\;0.69,\;0.56,\;0.35,\;0.24)^T\end{array}$$

According to Eq. 3:

$$\left( {R_{35},\;R_{40},\;R_{50},\;R_{60},\;R_{70}} \right) = C_{5 \times 5} \cdot \left( {S_{35},\;S_{40},\;S_{50},\;S_{60},\;S_{70}} \right)$$

As a demonstration, we simply obtain the conversion matrix through matrix operations. The solution of *C*_5×5_ is:

$$C_{5 \times 5} = \left( {\begin{array}{*{20}{l}} {12.67,} \hfill & { - 23.51,} \hfill & {7.67,} \hfill & {23.28,} \hfill & { - 26.34} \hfill \\ {40.33,} \hfill & { - 58.00,} \hfill & {7.46,} \hfill & {64.51,} \hfill & { - 74.37} \hfill \\ {18.67,} \hfill & { - 38.04,} \hfill & {21.04,} \hfill & {27.65,} \hfill & { - 33.83} \hfill \\ {35.83,} \hfill & { - 57.00,} \hfill & {15.27,} \hfill & {81.74,} \hfill & { - 97.65} \hfill \\ {17.33,} \hfill & { - 34.50,} \hfill & {18.08,} \hfill & {36.14,} \hfill & { - 46.39} \hfill \end{array}} \right)$$

The inverse matrix of the conversion matrix is,

$$C_{5 \times 5}^{ - 1} = 10^{ - 2} \times \left( {\begin{array}{*{20}{l}} { - 66.43,} \hfill & {3.91,} \hfill & {41.56,} \hfill & {18.22,} \hfill & { - 37.18} \hfill \\ { - 49.82,} \hfill & { - 3.89,} \hfill & {33.70,} \hfill & {20.98,} \hfill & { - 34.21} \hfill \\ { - 31.18,} \hfill & { - 6.73,} \hfill & {30.34,} \hfill & {15.99,} \hfill & { - 27.28} \hfill \\ {4.25,} \hfill & { - 15.87,} \hfill & {33.87,} \hfill & {26.18,} \hfill & { - 56.77} \hfill \\ {3.39,} \hfill & { - 10.63,} \hfill & {28.67,} \hfill & {17.83,} \hfill & { - 45.47} \hfill \end{array}} \right)$$

Then we could use the above-derived matrix to discriminate the unknown X-ray spectra. The Validation Datasets were obtained by setting the driving voltages of the X-ray tube at 45, 55, and 65 kVp and collecting the response matrix *R*_5×3_ at the same time. Then the spectral matrix *S*_5×3_ could be reconstructed by pre-multiplying the response matrix by the inverse matrix of the *C*_5×5_, as Eq. 6:

$$C_{5 \times 5}^{ - 1} \cdot R_{5 \times 3} = S_{5 \times 3}$$

**Fig. 4:**
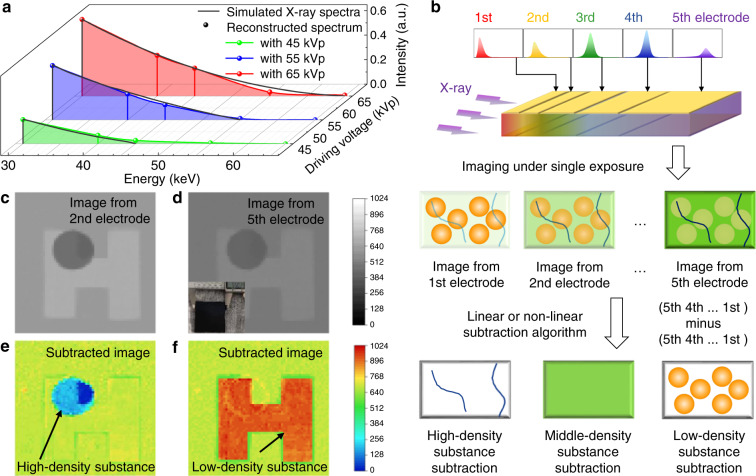
The applications of the multi-energy X-ray detector. **a** The reconstructed X-ray spectra obtained from the response signals of the detector, and the simulated X-ray spectra by Monte Carlo, when the driving voltages of the X-ray tube are 45, 55, 65 kVp. **b** Schematic diagram of multi-energy X-ray imaging. The X-ray energy from low to high is represented by the color from red to purple. The dark-blue lines represent high-density substance, yellow-green rectangles represent medium-density substance and orange balls represent low-density substance. **c** The image of our artificial sample from 2nd electrode. **d** The image from 5th electrode. The inset is the optical photo of the sample. **e** The subtracted pseudocolor image for high-density substance CaCO_3_ tablet. **f** The subtracted image obtained for the low-density paraffin ‘H’ letter. The pixel size in **c**–**f** is 0.85 mm

The elements of the reconstructed spectral matrix, as sampling points, are shown in Fig. 4a. They not only have clear cut-off edges that match the actual driving voltages, but also have small gaps with the simulated X-ray spectral intensities represented by the black line, that is, the Loss Function is 10.41% (45 kVp), 2.77% (55 kVp), and 2.97% (65 kVp) respectively. This good match verifies the effectiveness of the conversion matrix. It could be expected that more pairs of electrodes could provide more information about the continuous multi-energy spectra.

At last, we used the detector for multi-energy X-ray imaging applications. In conventional dual-energy X-ray imaging, two different X-ray sources are used, or the driving voltage of the same tube is switched, in order to provide two different X-ray spectra. Two sets of images are obtained under two times exposure, and the image containing only low-density tissue and the image containing only high-density tissue can be obtained through subtraction algorithms^11^. Here, we detected an artificial sample to further verify the feasibility of our novel detector for multi-energy X-ray imaging. The whole detection is under single exposure, as shown in Fig. 4b. In the schematic diagram Fig. S8, black, gray, and white colors represent the high-density, medium-density and low-density materials of the artificial sample, corresponding to the three most important components in the human body, skeletal (wet wt% ~10%)^29^, blood and muscle (about 1.0 g cm^−3^)^30^, and fat (wt% 10% to 20% for normal men, 0.9 g cm^−3^)^31^. During the production of the 10-mm-thickness artificial sample, we proportionally used CaCO_3_ (2.711 g cm^−3^), PDMS (about 1.0 g cm^−3^ at 20 °C), and paraffin (0.820 g cm^−3^) with similar densities to replace the above three substances respectively, in order to simulate the imaging process of human bodies. In addition, a little carbon black was added, so that the spatial distribution and the stacking situation of the sample inside could not be observed from outside, as illustrated in Fig. S8b. More information about the artificial sample and human tissues is shown in Supporting Information.

The images in Fig. 4c, d show the original dual-energy images from the 2nd and 5th electrodes for the artificial sample, respectively. The size of the imaged object is ~5.5 × 5.5 cm^2^ and the pixel size is 0.85 mm. Here we achieved the imaging by recording the signals from the five pixels along the incident direction of X-ray, and the imaging object was driven by X-Y motion stage. The images from 64 × 64 pixels arrays have been normalized to 1024 gray values, and they are not optimized by any algorithms. The image detected by the 2nd electrode contains more low-energy X-ray photons, while that detected by the 5th electrode contains more high-energy X-ray photons. Images in Fig. S9 are results of different-density-tissue subtraction and are obtained by various subtraction algorithms. Specifically, we take the logarithm of the gray values and then use a linear subtraction algorithm with the parameter *к*_H_ /*к*_L_ of 1.5, to obtain the image Fig. S9a, which emphasizes hard-density tissues. Then, we redraw it into a pseudocolor image as Fig. 4e. We further use high-order non-linear algorithms to optimize the effects of subtraction and obtain the image of objects as Fig. 4f. The orange letter ‘H’ in it does not overlap with other unconcerned tissues and it successfully demonstrates the result of soft-tissue subtraction. More information about the mathematical principles and parameter selection of subtraction processing can be found in the Supporting Information. Overall, the vertical matrix detector can effectively achieve multi-energy X-ray imaging under single exposure, not limited to dual X-ray sources.

## Discussion

This work raises a novel strategy of combining the vertical matrix perovskite detector with machine learning algorithm for multi-energy X-ray discrimination. The fabricated detector array based on MAPbBr_3_ single crystal exhibits good linear response and high sensitivity. The conversion matrix of the detector could be obtained by multiple training processes. We experimentally verified the effectiveness of above detectors in unknown X-ray spectrum reconstruction, and the cut-off edges accurately match with the actual spectrum. More importantly, we achieve multi-energy X-ray imaging under single exposure, which has not been achieved in traditional detectors. With the aid of subtraction method, we could clearly distinguish objects with low and high densities. We expect a similar effect could be achieved for other radiation detection semiconductors like CdZnTe. In sum, this work shifts the paradigm of multi-energy X-ray detection and brings encouraging opportunities for next-generation lowcost X-ray spectrometer and energy-resolved X-ray imaging.

## Materials and methods

### Materials

Anhydrous dimethylformamide (DMF) and absolute ethanol (≥99.7%) were obtained from Sinopharm. CaCO_3_ (99%, C111980) and Paraffin with ceresin (CAS 8002-72-2) were purchased from Aladdin. CH_3_NH_3_Br (MABr, 99.9%), PbBr_2_ (99.999%), and C_60_ (99.9%) were purchased from Advanced Election Technology Co. Ltd. Polydimethylsiloxane (PDMS) was obtained from Sigma–Aldrich. All chemicals were used without further purification.

### Crystal growth procedure

We grew the MAPbBr_3_ single crystal by the inverse temperature crystallization (ITC) method. MABr and PbBr_2_ with a molar ratio of 1.2:1 were dissolved into the DMF solution at a concentration of 1 mol L^−1^. The precursor solution was poured into a Teflon cup. Then, a seed crystal was added, and the Telflon cup was sealed with tin foil. The cup was placed in an oven. The solution was kept at 70 °C for 10 h. The obtained crystal has a size of 5 mm × 5 mm × 2 mm.

### Device fabrication

To obtain parallel surfaces, one face of the crystal was fixed onto the sample holder by paraffin wax, and then we polished the crystal by carefully controlling the holder. The crystal was polished with sandpaper and polishing pads. The final thickness is ~1 mm. 60-nm-thick Au electrodes were evaporated on one side, and the opposite side was thermally evaporated with 10-nm C_60_ and 80-nm Ag. The patterned electrodes were prepared by laser marking (light source: semiconductor laser, JPT YDFLP-20-LP1^+^-S). The average power was 20 W, the pulse width was 200 ns, and the center wavelength was 1064 nm. During laser marking, the processing speed was 200 mm s^−1^, the laser power was 5% and the frequency was 30 kHz. Here we used a self-made glass parallel track with scale to guarantee the alignment.

### Detector performance measurement

We used Keithley 6517B Source Meter to apply bias voltages and record the response current. For X-ray detection performance, Amptek Mini-X2 tube (Newton Scientific M237, Au target) was used as the X-ray source. Detailed parameters can be seen in Table S4. The dose rate had been calibrated with the ion chamber dosimeter (Magic Max from IBA Dosimetry). All measurement about X-ray detection was conducted in a dark lead box to minimize interference from ambient light and noise response.

### Monte Carlo simulation

pyPENELOPE was used for Monte Carlo simulation to obtain the X-ray spectrum under given driving voltages of X-ray tubes^32^. During simulation, the parameters, including electron beam energy, anode target, inclination angle, and filtering material, should be provided as Table S6.

### Preparation of the artificial sample for multi-energy X-ray imaging

The main inorganic component in bone is CaCO_3_ (2.711 g cm^−3^) and Ca_3_(PO_4_)_2_ (3.140 g cm^−3^). The density of muscle is about 1.060 g cm^−3^. The density of body fat is about 0.900 g cm^−3^. Here we used CaCO_3_, PDMS (1.0 g cm^−3^ at 20 °C), and paraffin (0.820 g cm^−3^) to mimic bones, muscles and body fat, respectively. According to the density from high to low, they are shown as black, gray and white in the Fig. S8. The CaCO_3_ tablet has a diameter of 13 mm. The large block of paraffin was fabricated into the shape of the letter ‘H’ by a wire cutting machine. We prepared 30 g PDMS solution with a mass ratio of prepolymer and curing agent of 10:1, and mixed 5 g carbon black powder. The carbon black was used to obscure views, so that the spatial distribution and the stacking situation of the artificial sample inside could not be observed from outside. The CaCO_3_ tablet and paraffin block were placed in a container, in which the carbon-PDMS mixture was poured. Finally, we put the container on the hot table at 80 °C for 1 h to make the mixture cured and got the black artificial sample with a size of 55 mm × 55 mm × 10 mm.

## Supplementary information

Supplementary Information for Vertical matrix perovskite X-ray detector for effective multi-energy discrimination

## Data Availability

The data that support the findings if this study is available from the corresponding authors upon reasonable request.
