# Supplementary material for: Vertical matrix perovskite X-ray detector for effective multi-energy discrimination
Source: Light Sci Appl. 2022 Apr 21;11:105. doi: 10.1038/s41377-022-00791-y (PMC9023493; doi:10.1038/s41377-022-00791-y)
Supplement: Supplementary file 1 — Supplementary Information for Vertical matrix perovskite X-ray detector for effective multi-energy discrimination [file 41377_2022_791_MOESM1_ESM.docx]

***Supplementary Information for***

**Vertical matrix perovskite X-ray detector for effective multi-energy discrimination**

Jincong Pang^1#^, Shan Zhao^1#^, Xinyuan Du^1^, Haodi Wu^1^, Guangda Niu^1,2*^, Jiang Tang^1,2^

1. Wuhan National Laboratory for Optoelectronics and School of Optical and Electronic Information, Huazhong University of Science and Technology, Wuhan 430074, China.

2. Optical Valley Laboratory, Wuhan 430074, China.

^#^ The authors contributed equally to this work.

Email: guangda_niu@hust.edu.cn

**Note I. Comparison of the energy discrimination capability for dual-layer-electrode and quintuple-layer-electrode detectors.**

To compare the energy discrimination ability of dual-layer detectors and quintuple-layer detectors, we calculated the theoretical signal difference in the energy range of 20 keV -70 keV at equal intervals of 10 keV in **Table S1** and **S2**. For a certain single energy X-ray, we normalized all response to the maximum response of each electrode and defined the electrode response with the largest difference as the energy discrimination between the two energy. A greater discrimination means that the electrode signal and the gray value of the image can have a greater difference. The electrode width of the quintuple-layer detector is the same as manufacturing thickness in **Table S3**, and the electrode width of the two-layer detector is calculated by the same algorithm documented in the article, where the thickness of the 1st electrode is 0.4 mm and the thickness of the 2nd electrode is 1.9 mm. As shown in **Table S1** and **Table S2**, the energy discrimination of the quintuple-layer detector is much higher than that of the double-layer.

**Note II. The principle of dual-energy and multi-energy subtraction process.**

The attenuation trend of X-ray has been described in **Equation 1**. For complex matter composed of multipe materials, it can be expressed by the integral of the material in a certain straight-line direction:

$$T=T_{0}e^{-\int\alpha(E, l)dl}$$

Most objects to be detected are composed of only a limited number of materials. If monochromatic X-ray is used at this time, *α*(*E*)=*α* is a fixed value. Then the logarithmic operation on the result of detection (*D*) can be simplified as:

$$D=-log \frac{T}{T_{0}}=\sum_{1}^{p} \alpha_{p}l_{p}+\mathrm{Const}$$

We can see that the detection signal is actually determined by the attenuation coefficient of the materials to the X-ray. Given that the object has only two materials with different densities,^1^ we can get high-energy signal, *D*_H_, and low-energy signal, *D*_L_:

$$D_{H}=\alpha_{1H}l_{1}+\alpha_{2H}l_{2}+\mathrm{Const}_{H}$$

$$D_{L}=\alpha_{1L}l_{1}+\alpha_{2L}l_{2}+\mathrm{Const}_{L}$$

Multiply weighting coefficients, *к*, to the above two detection signals to obtain the subtraction signal:

$$D_{\mathrm{sub}}=\kappa_{H}D_{H}-\kappa_{L}D_{L}=l_{1}(\kappa_{H}\alpha_{1H}-\kappa_{L}\alpha_{1L})+l_{2}(\kappa_{H}\alpha_{2H}-\kappa_{L}\alpha_{2L})+Const'$$

When the weighting coefficients, *к*_H_, *к*_L_ are appropriate values, the coefficient of *l*_1_ will be zero, that is, the detection result has nothing to do with the first material, but only with the second material. In the same way, changing the weighting coefficients can also make the result independent from *l*_2_.

In traditional dual-energy detection, due to the broad-spectrum characteristics and beam hardening effect of the nearly monochromatic X-ray, people need to use algorithms to correct the non-linear characteristics of the absorption coefficient.^2,3^ In the article, we used continuous multi-energy X-ray, rather than nearly monochromatic X-ray, which makes the absorption coefficient as a function of energy. Here we use second-order polynomial fitting to improve the subtraction algorithm:^1,4^

$$D_{H}=\beta_{0}+\beta_{11}l_{1}+\beta_{12}{l_{1}}^{2}+\beta_{21}l_{2}+\beta_{22}{l_{2}}^{2}+{\beta'}_{12}l_{1}l_{2}+\mathrm{Const}_{H}$$

$$D_{L}=\varepsilon_{0}+\varepsilon_{11}l_{1}+\varepsilon_{12}{l_{1}}^{2}+\varepsilon_{21}l_{2}+\varepsilon_{22}{l_{2}}^{2}+{\varepsilon'}_{12}l_{1}l_{2}+\mathrm{Const}_{L}$$

Introducing the correction algorithm makes the coefficient terms not predetermined. But we can still get the specific coefficient values through mathematical analysis of the detection results of the actual imaging system. In addition, other high-order algorithms can also implement this process. In practice, the human body has a variety of tissues with different densities. But this does not affect the subtraction results. In the demonstration of the article, we use artificial sample with three different materials as an extension.

$D_{\mathrm{sub}}=(\beta_{0}-\varepsilon_{0})+(\beta_{11}-\varepsilon_{11})l_{1}+(\beta_{12}-\varepsilon_{12}){l_{1}}^{2}+(\beta_{21}-\varepsilon_{21})l_{2}+$

$$(\beta_{22}-\varepsilon_{22}){l_{2}}^{2}+(\beta_{31}-\varepsilon_{31})l_{3}+(\beta_{32}-\varepsilon_{32}){l_{3}}^{2}+({\beta'}_{12}-{\varepsilon'}_{12})l_{1}l_{2}+$$

$$({\beta'}_{13}-{\varepsilon'}_{13})l_{1}l_{3}+({\beta'}_{23}-{\varepsilon'}_{23})l_{2}l_{3}+Const'$$

$D_{\mathrm{sub}}={[\beta}_{11}-\varepsilon_{11}+({\beta'}_{12}-{\varepsilon'}_{12})l_{2}+({\beta'}_{13}-{\varepsilon'}_{13})l_{3}]l_{1}+.....{}_{{}}$There are parameters, *β*, *ε*, that make the coefficient term of thickness *l*_1_ zero.

**Note III. Crosstalk.**

We carefully studied the potential signal crosstalk within the vertical matrix electrodes. There are three physical processes causing interpixel crosstalk: i) interpixel charge diffusion, ii) photoelectron cloud dispersion, iii) re-absorption of k-edge fluorescence.

i) Interpixel charge diffusion.

In our prototype detectors, there is no interpixel charge diffusion, since we measured the response from the pixels one by one rather than applying external bias simultaneously on all pixels. The carriers within the pixel region would only be extracted by this pixel electrode. In future work for integration with back-end circuits, the interpixel charge diffusion could be suppressed by adding electric-field shield between pixels, as documented in our previous works (DOI: 10.1016/j.fmre.2021.10.004).

ii) Photoelectron cloud dispersion.

The incident X-ray will firstly excite the primary electron-hole pairs, and then gradually excite more secondary electron-hole pairs. By the end of the interaction process, a cloud of the electron-hole pairs is created. The range of photoelectrons is determined from the maximum range of the electron-hole pairs *R*_max_. The empirical expression of *R*_max_ was raised by Kanaya and Okayama (DOI: 10.1063/1.2990765):

$$R_{\max}=2.761\times{10}^{-6}\times\frac{M_{\mathrm{at}}{E_{0}}^{\frac{5}{3}}}{\rho Z^{\frac{8}{9}}}$$

where *ρ* is the material density, *M*_at_ is the average atomic mass, *Z* is the atomic number and *E*_0_ is the energy of the primary photoelectron. Using the interested energy in this paper (30-70 keV), we can estimate the maximum radius as 32.8 μm, which can be ignored compared to the channel width between pixelated electrodes (60 μm).

iii) Re-absorption of k-edge fluorescence.

After the photoelectric absorption of the incident X-ray, a characteristic K-fluorescent or L-fluorescent X-ray may be emitted, as the empty states left in the K or L shells are filled by an outer shell electron. For MAPbBr_3_, the K and L shell fluorescent energy of the elements are shown in **Table S7**.

When using the energy of 30-70 keV in the experiment, all generated characteristic X-ray is under 15 keV. It is estimated that 99.5% of the energy will be re-absorbed within 60 μm MAPbBr_3_. Thereby, K-edge fluorescence will not bring serious crosstalk.

**Table S1**. The theoretical energy discrimination capability for quintuple-layer detector.

| X-ray energy (keV) | Electrodes | | | | | Energy discrimination capability |
| --- | --- | --- | --- | --- | --- | --- |
|  | 1st | 2nd | 3rd | 4th | 5th |  |
|  | 0.10 mm | 0.15 mm | 0.20 mm | 0.35 mm | 1.50 mm |  |
| 20 | 1.000 | 0.087 | 0.002 | 1.509E-5 | 2.510E-9 |  |
| 30 | 1.000 | 0.535 | 0.168 | 0.035 | 0.002 | 44.8%  (30 keV-20 keV) |
| 40 | 1.000 | 0.921 | 0.620 | 0.384 | 0.127 | 45.2%  (40 keV-30 keV) |
| 50 | 0.875 | 1.000 | 0.911 | 0.887 | 0.739 | 61.2%  (50 keV-40 keV) |

Note: The energy discrimination capability compares the two sets of energy shown in parentheses.

**Table S2**. The theoretical energy discrimination capability for dual-layer detector.

| X-ray energy (keV) | Electrodes | | Energy discrimination capability |
| --- | --- | --- | --- |
|  | 1st | 2nd |  |
|  | 0.4 mm | 1.90 mm |  |
| 20 | 1.000 | 4.79E-5 |  |
| 30 | 1.000 | 0.034 | 3.4% (30 keV-20 keV) |
| 40 | 1.000 | 0.257 | 22.3% (40 keV-30 keV) |
| 50 | 1.000 | 0.700 | 44.3% (50 keV-40 keV) |

**Table S3**. The designed and measured thickness of the electrodes.

| Electrode number | 1st | 2nd | 3rd | 4th | 5th |
| --- | --- | --- | --- | --- | --- |
| Designed thickness (mm) | 0.109 | 0.138 | 0.215 | 0.364 | 1.531 |
| Measured thickness (mm) | 0.080 | 0.120 | 0.160 | 0.315 | 1.450 |

**Table S4**. The fitting linearity of the response signals to X-ray dose rates for **Figure 3**b.

|  | 1st | 2nd | 3rd | 4th | 5th |
| --- | --- | --- | --- | --- | --- |
| R^2^ | 0.99911 | 0.98981 | 0.99295 | 0.99250 | 0.99861 |

**Table S5**. The raw response current data of the vertical-matrix PSC device in **Figure 3**.

| Tube current | Electrodes | Response signals of X-ray spectrum (nA) | | | | |
| --- | --- | --- | --- | --- | --- | --- |
|  |  | 35 kVp | 40 kVp | 50 kVp | 60 kVp | 70 kVp |
| 140 μA | 1st | 1.03 | 1.46 | 2.13 | 2.86 | 3.55 |
|  | 2nd | 3.30 | 5.03 | 8.33 | 11.4 | 11.8 |
|  | 3rd | 3.04 | 2.46 | 4.27 | 6.26 | 10.1 |
|  | 4th | 2.98 | 4.30 | 7.12 | 9.94 | 13.3 |
|  | 5th | 1.41 | 1.90 | 3.24 | 5.11 | 7.15 |
| 120 μA | 1st | 0.89 | 1.24 | 1.84 | 2.47 | 3.06 |
|  | 2nd | 2.86 | 4.36 | 7.29 | 10.1 | 10.5 |
|  | 3rd | 2.72 | 2.15 | 3.71 | 5.43 | 8.65 |
|  | 4th | 2.57 | 3.73 | 6.24 | 8.79 | 11.7 |
|  | 5th | 1.23 | 1.64 | 2.79 | 4.40 | 6.16 |
| 100 μA | 1st | 0.76 | 1.05 | 1.54 | 2.08 | 2.57 |
|  | 2nd | 2.42 | 3.68 | 6.24 | 8.66 | 9.22 |
|  | 3rd | 1.40 | 1.84 | 3.14 | 4.60 | 7.24 |
|  | 4th | 2.15 | 3.16 | 5.36 | 7.64 | 10.2 |
|  | 5th | 1.04 | 1.39 | 2.34 | 3.68 | 5.17 |
| 80 μA | 1st | 0.63 | 0.86 | 1.25 | 1.69 | 2.08 |
|  | 2nd | 1.98 | 3.01 | 5.20 | 7.27 | 7.94 |
|  | 3rd | 1.38 | 1.53 | 2.58 | 3.77 | 5.83 |
|  | 4th | 1.74 | 2.59 | 4.48 | 6.49 | 8.71 |
|  | 5th | 0.85 | 1.13 | 1.88 | 2.97 | 4.18 |
| 60 μA | 1st | 0.49 | 0.67 | 0.95 | 1.30 | 1.59 |
|  | 2nd | 1.54 | 2.33 | 4.16 | 5.88 | 6.65 |
|  | 3rd | 1.17 | 1.22 | 2.01 | 2.94 | 4.41 |
|  | 4th | 1.32 | 2.02 | 3.61 | 5.34 | 7.19 |
|  | 5th | 0.67 | 0.87 | 1.43 | 2.26 | 3.18 |
| 40 μA | 1st | 0.37 | 0.48 | 0.66 | 0.91 | 1.10 |
|  | 2nd | 1.09 | 1.66 | 3.11 | 4.49 | 5.37 |
|  | 3rd | 0.95 | 0.91 | 1.45 | 2.11 | 3.00 |
|  | 4th | 0.91 | 1.45 | 2.73 | 4.20 | 5.68 |
|  | 5th | 0.48 | 0.61 | 0.98 | 1.54 | 2.19 |
| 20 μA | 1st | 0.24 | 0.29 | 0.37 | 0.52 | 0.61 |
|  | 2nd | 0.65 | 0.99 | 2.07 | 3.10 | 4.09 |
|  | 3rd | 0.73 | 0.60 | 0.89 | 1.27 | 1.59 |
|  | 4th | 0.49 | 0.88 | 1.85 | 3.05 | 4.16 |
|  | 5th | 0.29 | 0.35 | 0.53 | 0.83 | 1.20 |

**Table S6**. The setting parameters of X-ray tube for Monte Carlo simulation.

| Parameter | Settings |
| --- | --- |
| target material | Au |
| target inclination angle | 12° |
| filter material and thickness | Al, 2 mm |
| Driving conditions | Voltage from 35 to 70 kVp  Current from 10 to 140 μA |
| Maximum output power | 10 W |

**Table S7**. The K shell and L shell fluorescent energy of all elements in MAPbBr_3_.

| element | K_α1_  (keV) | K_α2_  (keV) | K_β1_  (keV) | K_β2_  (keV) | L_α_  (keV) | L_β_  (keV) | L_γ_  (keV) |
| --- | --- | --- | --- | --- | --- | --- | --- |
| C | 0.282 | |  | | | | |
| N | 0.392 | |  | | | | |
| Pb | 74.957 | 72.794 (55%) | 84.922 (35%) | 87.343 (10%) | 10.549 10.448 | 12.611 12.620 | 14.762 |
| Br | 11.923 | 11.877 (50%) | 13.290 (21%) | 13.465 (1.7%) | 1.480 | 1.526 |  |

**
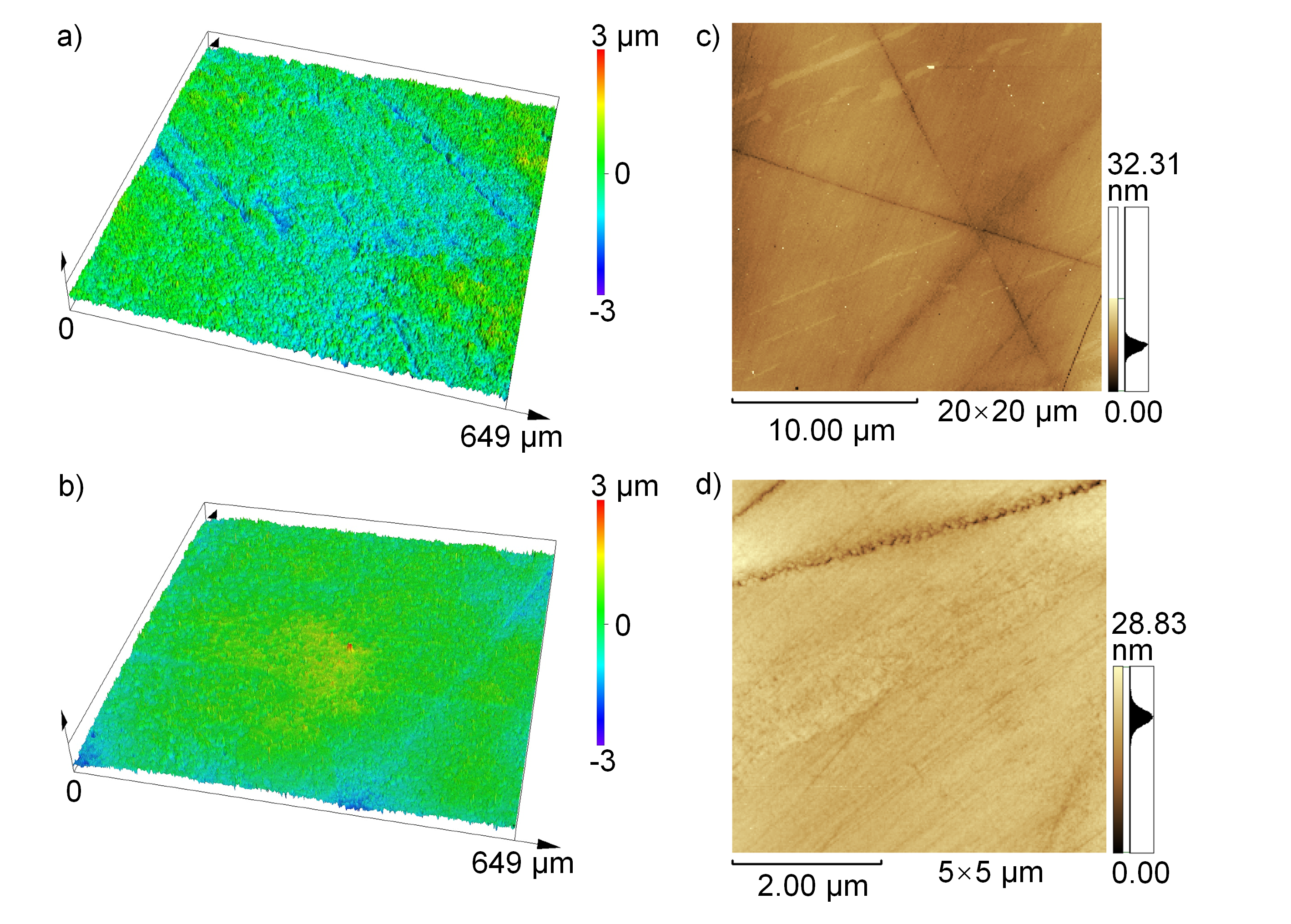
**

**Figure S1.** a) and b) Scanning laser confocal microscopy pictures of the front and back surfaces of the crystal. c) and d) Atomic force microscope pictures of the front and back surfaces. Average roughness *R*_a_ is 1.618 nm for (c) and 1.245 nm for (d).


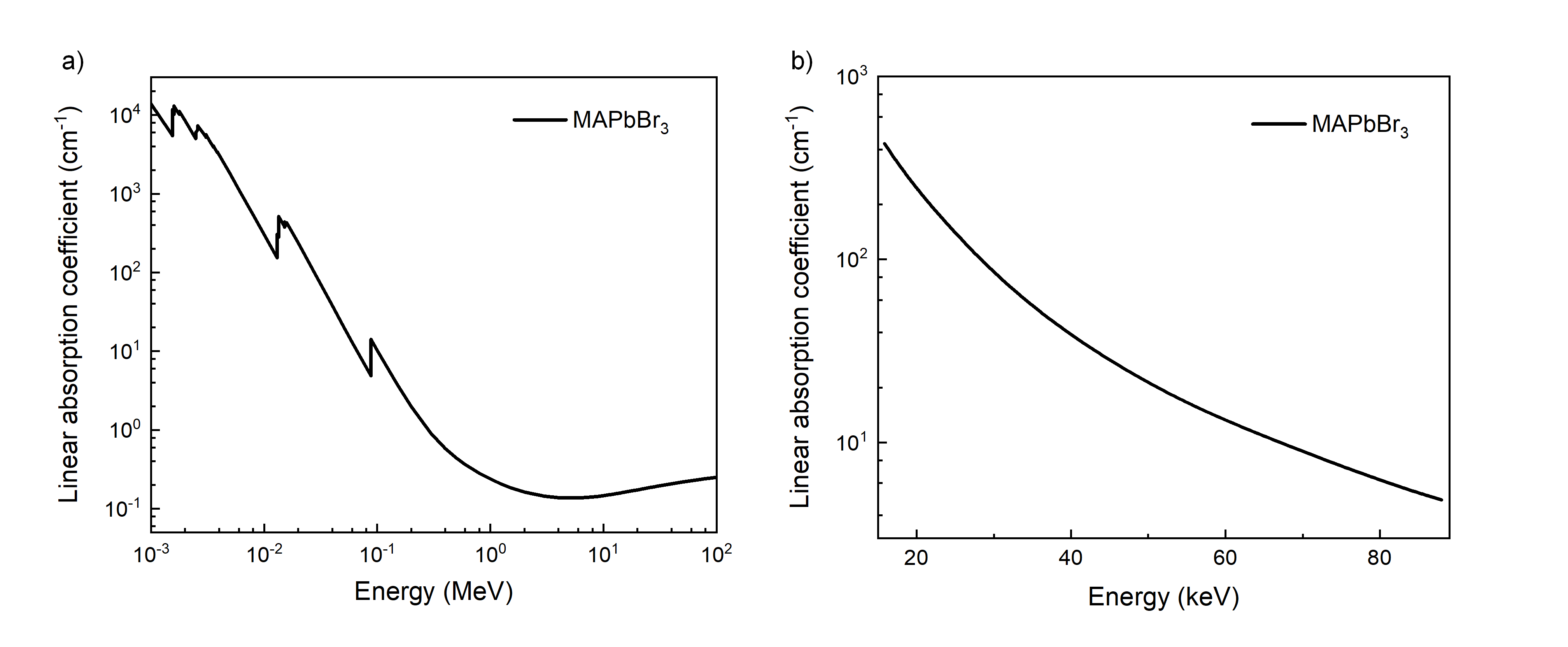


**Figure S2.** a) The linear attenuation coefficient of MAPbBr_3_ under a wide range of photon energy (1 keV to 100 MeV). b) The attenuation coefficient for the specific energy range from 30 to 70 keV.

**
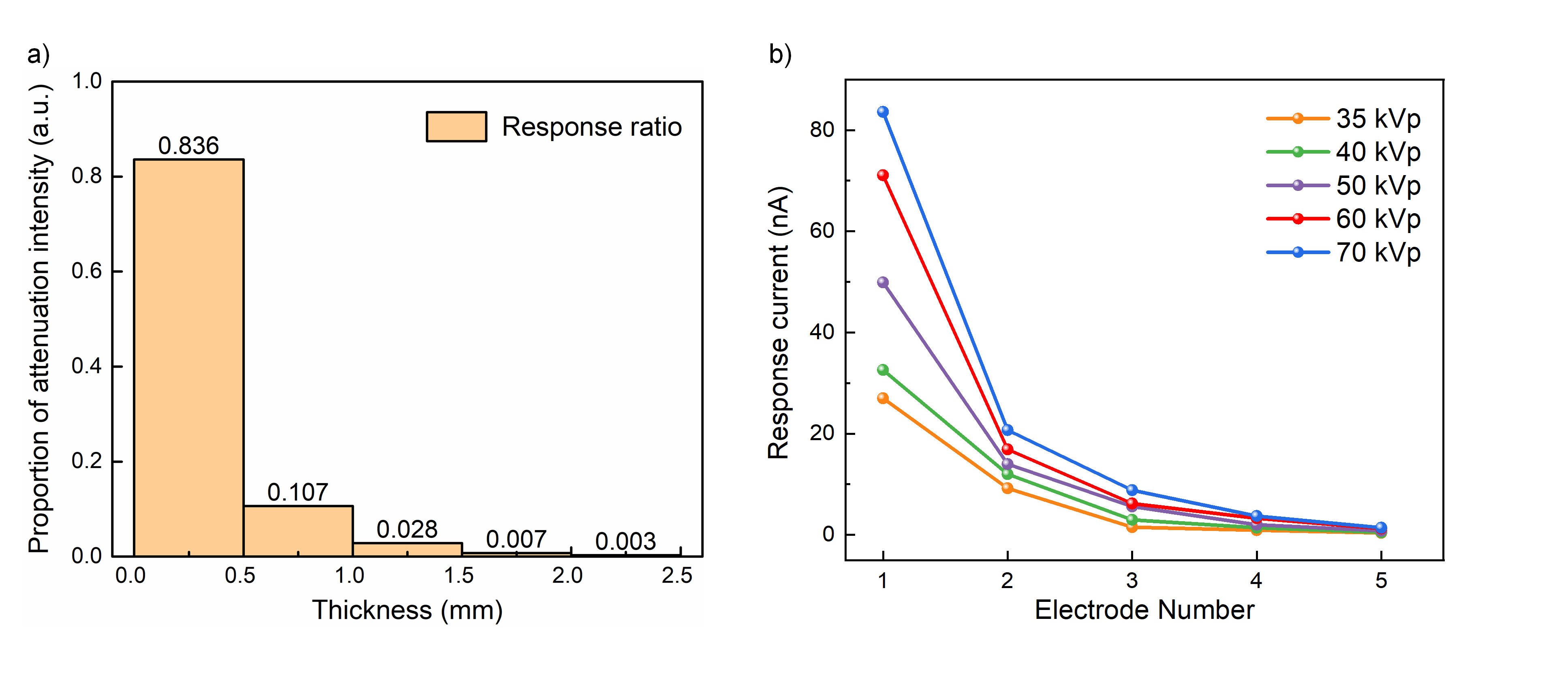
**

**Figure S3.** a) The theoretical proportion of X-ray intensity attenuated by the five strip electrodes with equal thickness (500 μm). b) The actual response current of the five electrodes with equal thickness. Since most energy was deposited on the shallow layers of the detector, the bottom detector (high electrode number) exhibited low response, which could be easily submerged in the signals of the adjacent electrodes.


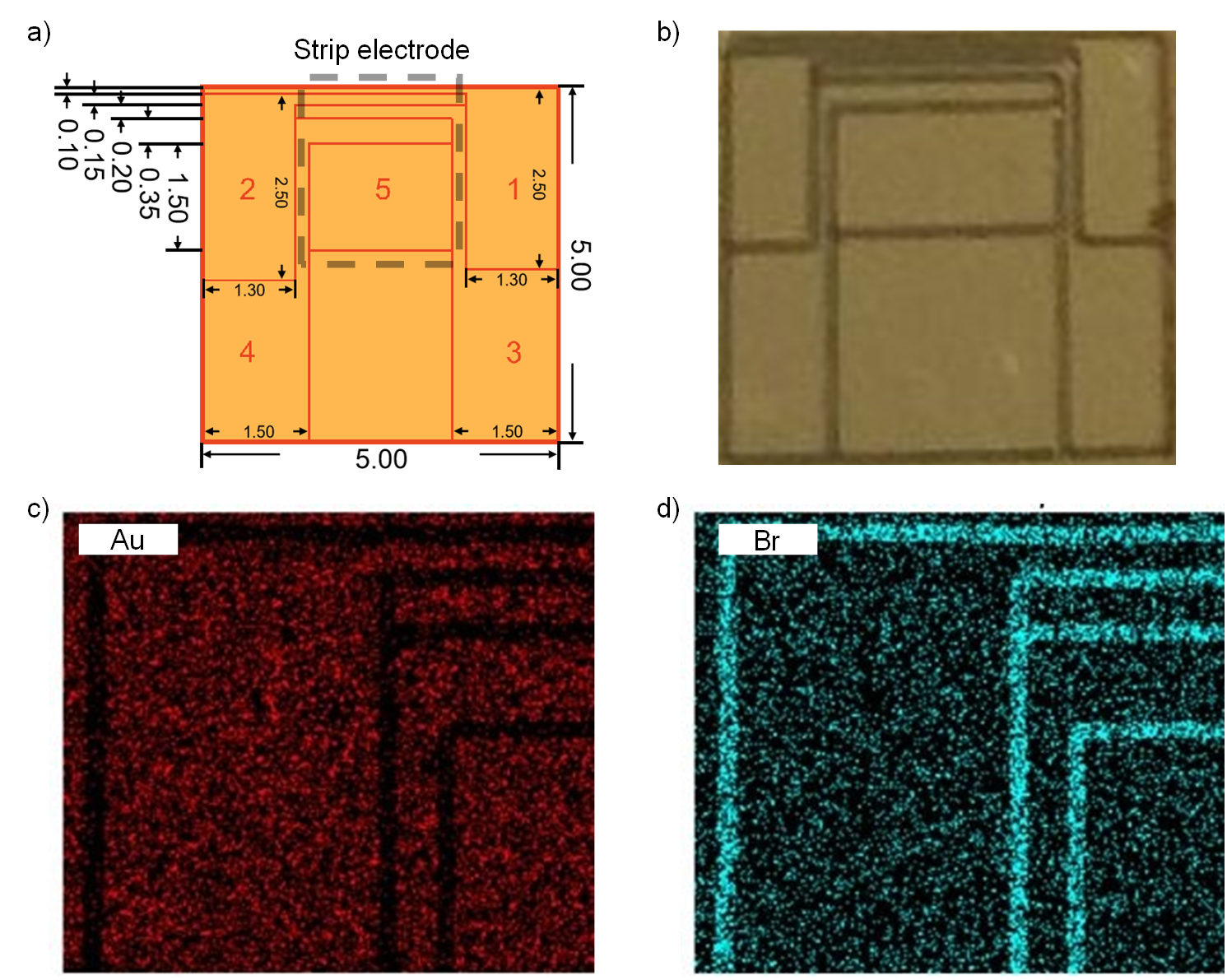


**Figure S4.** a) The drawing of the laser beam processing. b) The optical photo of the fabricated electrode pattern. c) and d) are energy dispersion spectrum scanning images of Au element and Br element, respectively.

**
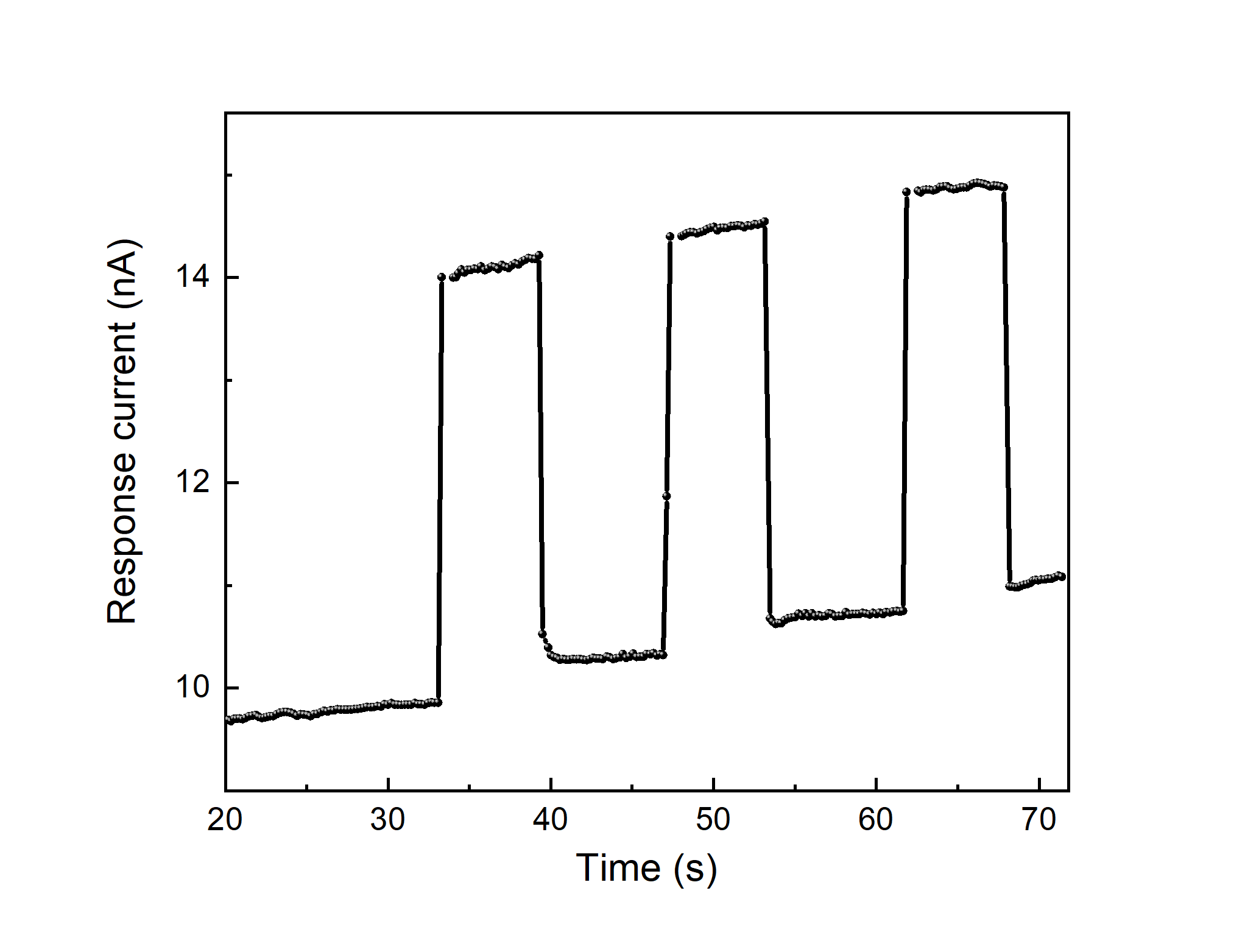
**

**Figure S5.** The response current of the Au-PSC-Ag device without C_60_.

**
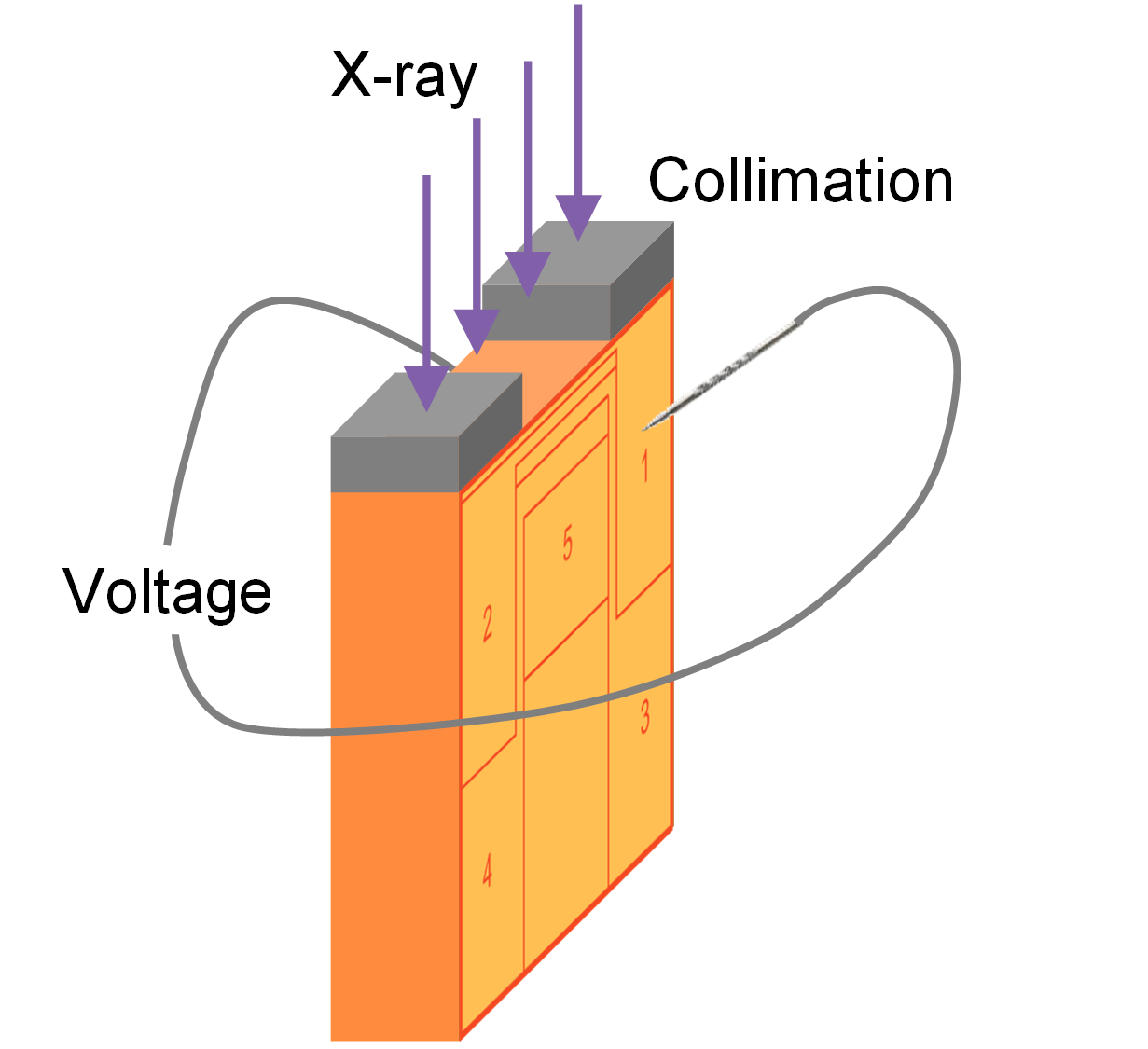
**

**Figure S6.** Schematic diagram of the testing process. X-ray is incident from the side of the device, and is firstly attenuated by the uppermost layer, *i.e.* the 1st electrode. The lead-out parts are blocked by the collimation lead sheets to shield X-ray, which avoids interference.


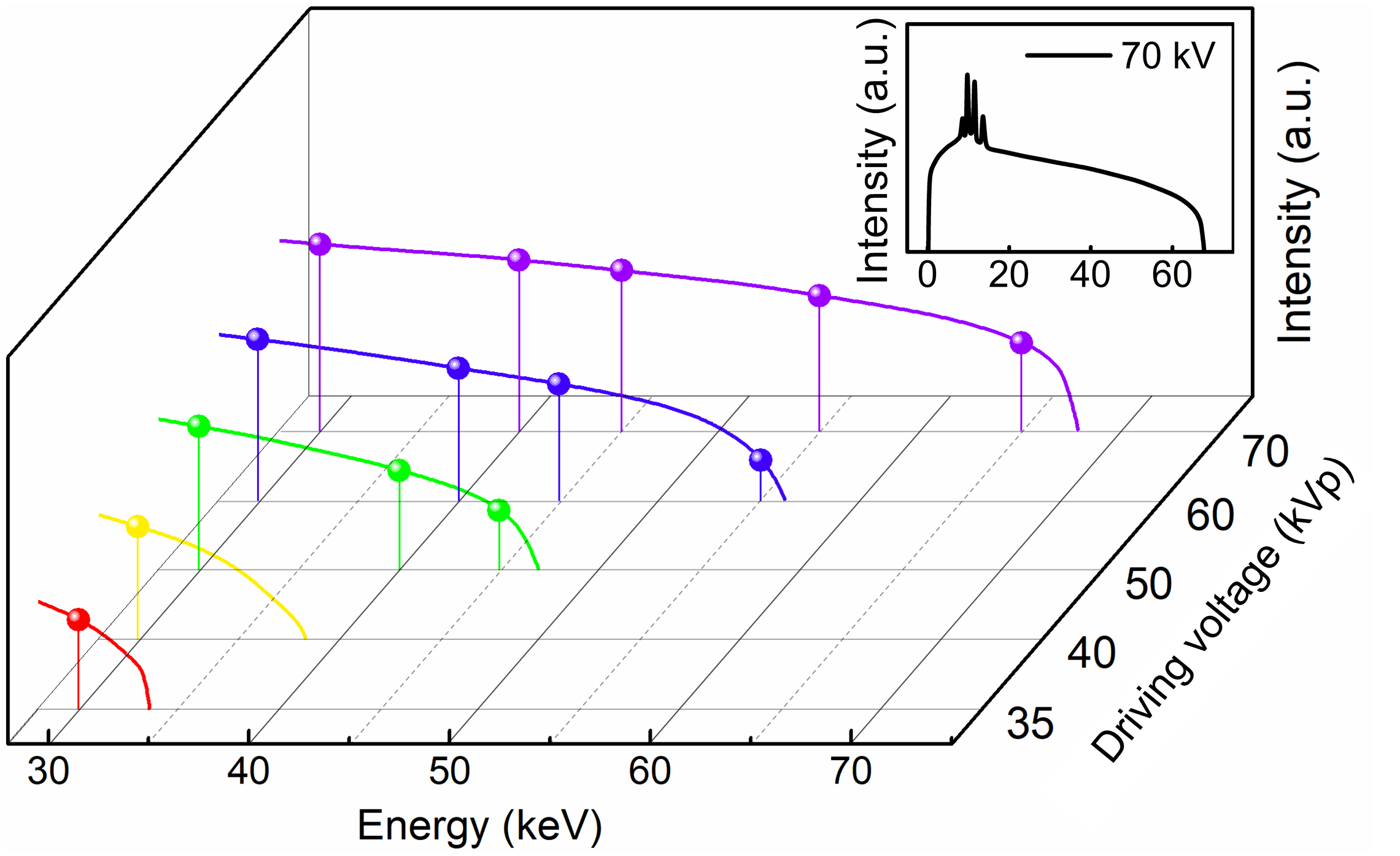


**Figure S7.** X-ray spectra on the logarithmic axis obtained by Monte Carlo simulation. The driving voltages are 35, 40, 50, 60, and 70 kVp, and the sampling points are located at 30, 40, 45, 55 and 65 keV, respectively. The illustration shows the entire spectrum, including the characteristic peaks of the anode target and the low energy parts without sampling points. The sampling points do not fall on characteristic peaks area.

**
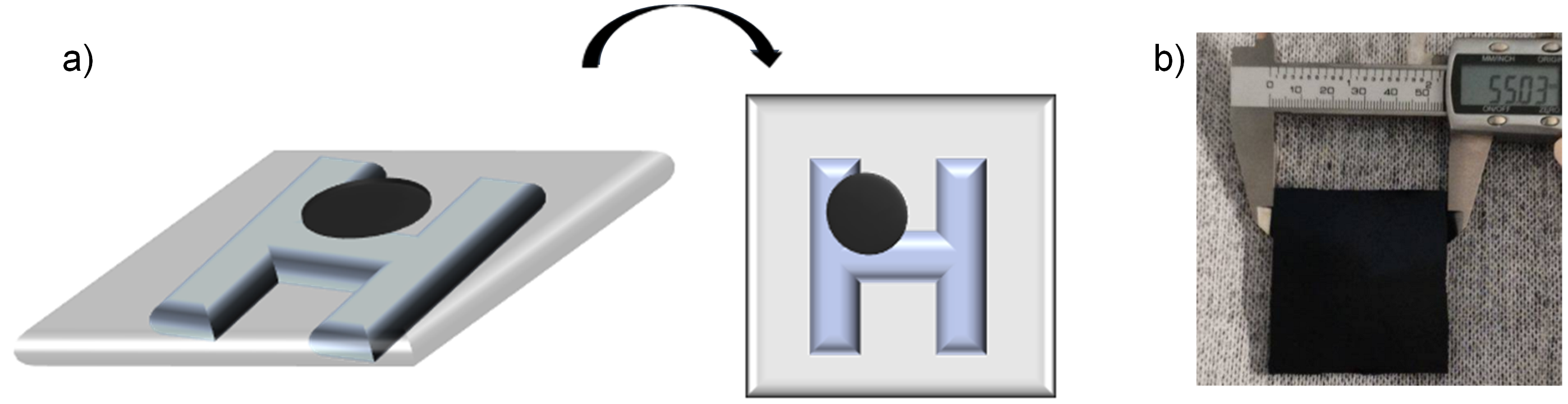
**

**Figure S8.** (a) Schematic illustration of the artificial sample. It is composed of three substances, and the substances are represented as black circle, gray square and white ‘H’ letter according to the density from high to low. These three substances are CaCO_3_, PDMS and paraffin, respectively. (b) Photo image of the artificial sample.


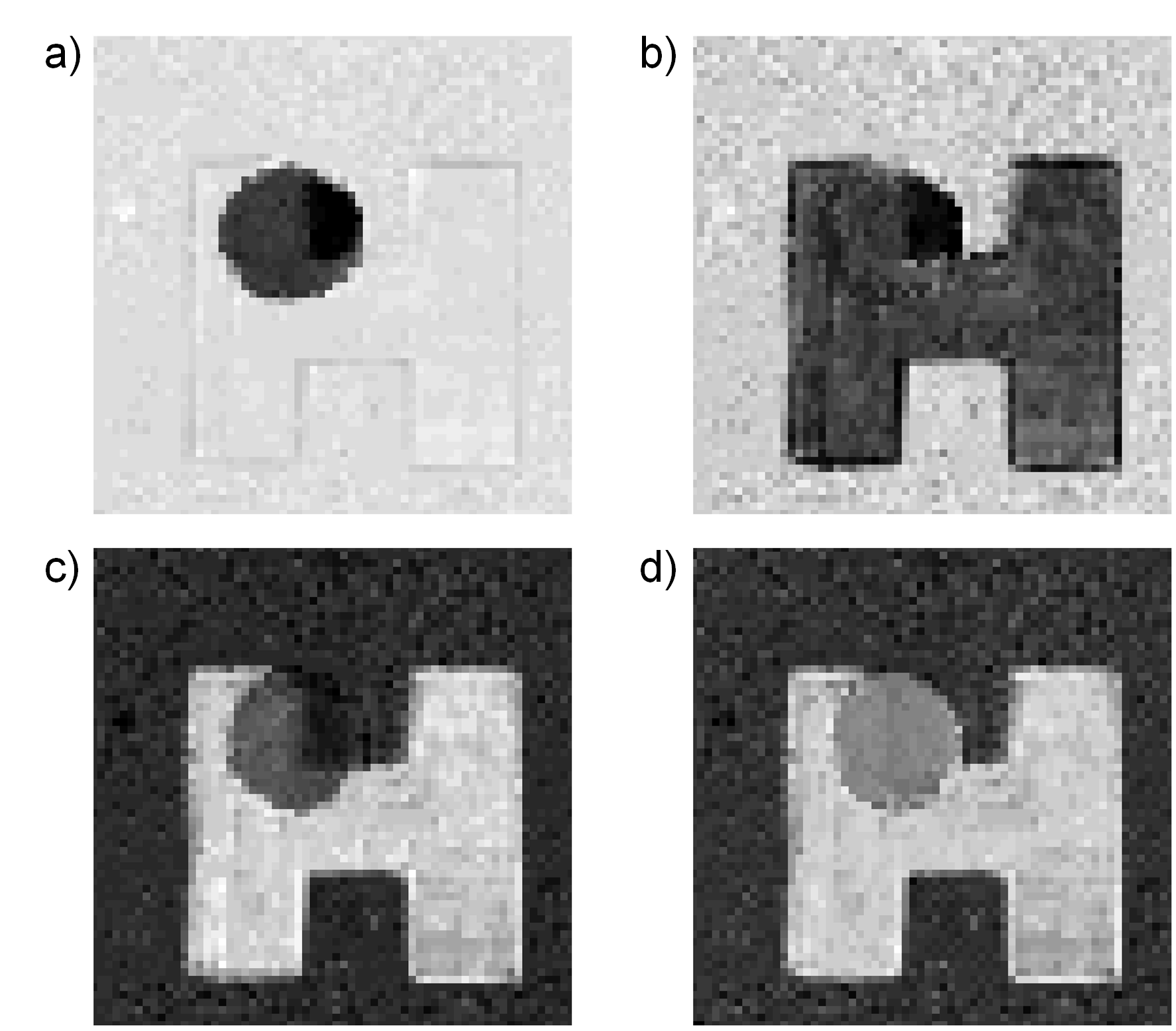


**Figure S9.** The grayscale images processed by the linear subtraction algorithms with different parameters. Specially, the parameter *к*_H_ /*к*_L_ is 1.5, 2.0, 2.35 and 2.46 from (a) to (d), respectively.

**References:**

1. Rebuffel, V. & Dinten, J. M. Dual-energy X-ray imaging: benefits and limits. *Insight - Non-Destructive Testing and Condition Monitoring* **49**, 589-594 (2007).
2. Cardinal, H. N. & Fenster, A. An accurate method for direct dual-energy calibration and decomposition. *Medical physics* **17**, 327-341 (1990).
3. Goh, K. L., Liew, S. C. & Hasegawa, B. H. Correction of energy-dependent systematic errors in dual-energy X-ray CT using a basis material coefficients transformation method. *IEEE Transactions on Nuclear Science* **44**, 2419-2424 (1997).
4. Létang, J. M. Freud, N. & Peix, G. Signal-to-noise ratio criterion for the optimization of dual-energy acquisition using virtual x-ray imaging: application to glass wool. *Journal of Electronic Imagin*g **13**, 436-449 (2004).
